# Correlating Radiographic Fractal Analysis at Implant Recipient Sites with Primary Implant Stability: An In Vivo Preliminary Study

**DOI:** 10.7759/cureus.6539

**Published:** 2020-01-02

**Authors:** Elie Hayek, Georges Aoun, Roger Bassit, Ibrahim Nasseh

**Affiliations:** 1 Oral and Maxillofacial Radiology, Faculty of Dental Medicine, Lebanese University, Beirut, LBN; 2 Oral Surgery, Faculty of Dental Medicine, Lebanese University, Beirut, LBN

**Keywords:** trabecular bone, implant site, psp digital radiography, edentulous region, primary stability

## Abstract

Objectives

The aim of this study was to demonstrate a possible correlation between radiographic trabecular bone quantified with fractal dimensions analysis and values of primary implant stability deliberated using the Osstell^®^ monitor (Integration Diagnostics AB, Goteborgsvagen, Sweden) with the density of bone specimens harvested from the implant recipient sites and calculated using the mass and volume of the bone bloc specimens.

Material and methods

Fifty implants of the same brand, diameter, and length were inserted with the same surgical procedures; 25 implants in the molar premolar region of the maxilla and 25 implants in the mandibular posterior region of only healthy male patients between 20 and 50 years of age. Prior to the placement of the implants, biopsies were taken from the selected site for density calculation using a calibrated trephine. Two photostimulable phosphor digital periapical radiographs were obtained for each patient and two regions of interest were selected for a fractal dimension analysis: one site before implantation and the same site immediately postoperatively adjacent to the implant.

Results

There were correlations between the fractal dimensions and implant stability quotient as well as between fractal dimensions and bone density. No significant difference was found between the fractal dimensions of pre- and postoperative periapical radiographs for the same region of interest and between implant stability quotient values of the maxillae and mandible sites.

Conclusion

All executed analyses were helpful in assessing the bone density in the recipient site of implant placement using fractal dimensions, offering complementary information about predictable bone density assessed on a periapical radiograph.

## Introduction

The most important key factors for successful implant treatment, excluding the patient’s general health and the biocompatibility of the implant material, are the quality and quantity of the local bone. In their studies, Esposito et al. associated the low bone quality to the oral implant failures [1] and Shalabi et al. connected the implant’s stability, in addition to the implant’s design and the surgical technique, to the quality and quantity of the bone [2].

To analyze the bone quality, two noninvasive clinical methods are in use: a) the fractal dimension (FD) as a preoperative tool and b) the implant stability quotient (ISQ) as a postoperative tool.

The resonance frequency analysis (RFA) is a method presented by Meredith et al. [3] that measures the primary stability using the piezo effect to produce a deflection of the implant and to provide ISQ values on a scale of 0 to 100.

Another imperative factor for successful implant treatment is the quality of the bone; the fractal analysis is one of the techniques used to evaluate bone quality. In 1983, Mandelbrot brought the concept of 'fractals' and FDs have since made a major contribution to the description and measurement of morphology in the natural world [4]. They have been applied to describe cell outlines, pulmonary branching, and, especially, to analyze the trabecular bone architecture [5-7]. Since the fractal index is simple to be calculated from periapical radiographs of a bone in the edentulous region, it may prove useful in estimating bone strength [8]. For Jolley et al., FD is a noninvasive technique for the quantitative evaluation of geometric structures shown through an image characterized by a single number [9]. It can be applicable in panoramic [10-11] and periapical radiographs [12-13].

Bone biopsies, which were obtained during oral implant surgery for histomorphometric evaluation, were utilized by Trisi and Rao [14]. Mish et al. correlated the percentage of bony trabeculae over the total biopsy area with the bone scoring recorded during the drilling of the implant bed, based on the hand-felt perception of the drilling resistance [15].

This present histological study has been established to calculate bone density through the measurement of mass and volume ratio of bone biopsies harvested from the recipient site.

Moreover, this clinical study aimed to compare the FD analysis and primary stability quotient with the histological density of bone bloc harvested from the site of implant placement for a possible correlation between these factors and the estimation of bone density prior to implant insertion using intraoral photostimulable phosphor (PSP) digital radiographs. Therefore, the correlation between FD, primary stability quotient and histological bone density was evaluated.

## Materials and methods

This study was conducted at the Division of Oral Radiology, with the contribution of the Department of Oral and Maxillofacial Surgery. All participating patients were carefully informed about the treatment procedure and their consent was obtained.

The inclusion criteria involved only male patients with an age range between 20 and 50, requiring at least one implantation in the premolar/molar region of the maxillae or mandible with more than 10 mm of a residual alveolar bone crest height and more than 5 mm of residual bone crest width based on cone beam computed tomography (CBCT) images.

The exclusion criteria included patients:

- Under medications that may affect the metabolism of bone

- With systemic conditions such as diabetes mellitus and so on

- With alveolar bone lesions or having received any type of bone graft

- Smokers

Fifty identical implants were inserted and distributed equally into the maxillae and the mandible, choosing only the molar and premolar edentulous regions. The length and diameter of all implants were 10 mm and 4 mm, respectively.

Prophylaxis sessions were done prior to all the surgeries performed by the same surgeon in accordance with a standard surgical technique.

The patients underwent intraoral (PSP) radiographs at the preoperative stage and postoperatively immediately after implant insertion. All radiographs for concerning sites were taken with a radiographic positioning device to allow acquisition with maximum reproducibility. Images were stored on a personal computer and given to the investigator for FD calculations.

During implant site preparation, bone specimens were harvested with a trephine bur (Trepan Bur, 2.0 mm diameter, 7 mm long, Komet Dental. Gebr. Brasseler Gmbh &Co, Germany). One bone specimen cylinder was obtained, and the trephine sites were used for implant installation.

Immediately after inserting the implant, the Resonance Frequency Analyzer (Osstell, Integration Diagnostics AB, Goteborgsvagen, Sweden) was used to measure the stability of each fixture as ISQ values and as recommended by the manufacturer. The measurements were repeated twice for each direction to ensure reproducibility, and if any difference was observed, the lower value was registered.

The mass of the biopsy was measured by a scientific electronic balance (the volume was constant since the diameter and length of the biopsy were known). For the descriptive histological analysis, the parameter evaluated was the mass of the specimen; then the density of bone biopsy was calculated from the ratio of the mass and volume of the bone specimen based on the formula D=M/V, with a bone density unit g/cm^3^.

**Figure 1:**
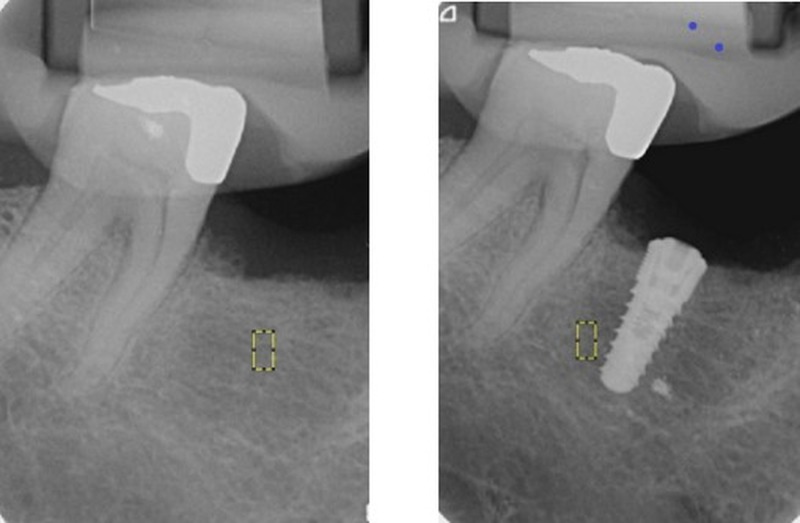
Region of interest at the recipient site on the preoperative radiograph and adjacent to the inserted implant immediately on postoperative radiograph - Preoperative periapical radiograph (before the insertion of the implant): a region of interest is created and set to a width of 25 pixels and a height of 50 pixels. The fractal dimension is then measured at this site. - Postoperative periapical radiograph (after the insertion of the implant): a region of interest is created with the same dimensions as those on the preoperative radiograph, adjacent to the inserted implant area. The fractal dimension is also measured at this site.

**Figure 2:**
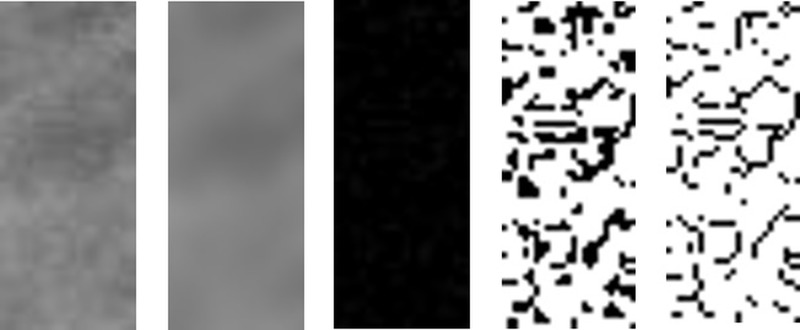
Image processing steps For fractal analysis, all 16-bit digital images are converted to 8-bit images. ImageJ® software is used for image processing and analyzing. The processing steps are as follows: a) the cropped ROI is duplicated by using the program menu of ImageJ® and saved as tiff; b) the duplicated image is then blurred with a Gaussian filter; c) the result of subtraction from the original image; d) the resultant image was converted to binary at the gray value of 128; e) finally, the image is skeletonized. The fractal dimension of this image is calculated using the box-counting function of the software. ROI: region of interest

The fractal dimensions were calculated using ImageJ software (version 1.36b, U.S. National Institutes of Health). A region of interest (ROI) was created, saved, and set to a width of 25 pixels and a height of 50 pixels. Two ROIs were selected on the radiographic images: area of the recipient site of the implant on the preoperative radiograph and adjacent to the inserted implant area on the postoperative radiograph (Figure 1). For FD analysis, ImageJ software was used for processing and analyzing all images (Figure 2).

All results were saved in an Excel sheet and all the data were statistically analyzed with the IBM® SPSS® software version 20.0 (SPSS Inc, Chicago, Illinois). The results showed that the data distribution was normal.

## Results

All patients were males with an age range between 20 and 50, with a mean age of 44.3 years. In this study, all implants were identical and no complications during the implant placement of any patient were mentioned.

Of the 50 implants placed, 25 implants were inserted in the posterior region of the maxilla and 25 implants in the posterior region of the mandible. In the maxilla, seven implants were installed in molar sites (three in the left side and four in the right), and 18 implants in premolar sites (12 in the left side and six in the right). In the mandible, among the 25 implants placed, 17 were in molar sites (11 in the right side and six in the left) and eight in premolar sites (two implants in the left side and six in the right). The distribution of the insertion of the implants is listed in Table 1.

**Table 1:** Distribution of implants' placement in the posterior region

			Molar (Right)	Molar (Left)	Premolar (Right)	Premolar (Left)	Total (Right)	Total (Left)
	Maxillae		4	3	6	12	10	15
	Mandibles		11	6	6	2	17	8
			15	9	12	14	27	23
Total		24			26		50	

In the histological analysis, it was observed that the bone density of the specimens differs between maxillary sites and mandibles sites. In some regions, the density of bone is higher than in others depending on the sites that the bone blocs were harvested (Table 2).

**Table 2:** Mean values of bone densities according to the sites of bone blocs

	Mean bone densities values (g/cm^3^)
Maxillary Molar	0.297
Maxillary Premolar	0.305
Mandibular Molar	0.379
Mandibular Premolar	0.489

In both maxillary and mandibular sites, a correlation was observed between FD and implant stability, as well as a statistically significant difference between the ISQ values of the maxillary region and mandibular region (Table 3).

**Table 3:** Comparison between ISQ mean values and fractal dimensions mean values ISQ: implant stability quotient; FD: fractal dimension

	Mean ISQ values	Mean FD values
Maxillary Molar	63.16	1.515
Maxillary Premolar	63.76	1.523
Mandibular Molar	72.35	1.550
Mandibular Premolar	70.16	1.563

The comparison between the ISQ values and the bone densities values showed a correlation between the maxillary and mandibular ISQs values and the bone densities of the similar sites, concluding the relationship between density of bone and implant primary stability (Table 4).

**Table 4:** Comparison between ISQ values and bone densities of similar sites ISQ: implant stability quotient

	Mean ISQ values	Mean bone densities values from different sites
Maxillary Molar	63.16	0.297
Maxillary Premolar	63.76	0.305
Mandibular Molar	72.35	0.379
Mandibular Premolar	70.16	0.489

The analysis of the mean value of FD on the preoperative radiograph and the mean value of FD on the postoperative radiograph showed no statistically significant correlation (Table 5).

**Table 5:** Comparison between FD values of pre and postoperative radiographs FD: fractal dimension

	Mean FD values of preoperative radiograph	Mean FD values of postoperative radiograph
Maxillary Molar	1.515	1.509
Maxillary Premolar	1.523	1.548
Mandibular Molar	1.550	1.574
Mandibular Premolar	1.563	1.562

Comparing the bone densities calculated from specimens harvested from different sites and FDs in all preoperative radiographs showed a positive correlation between FD and bone density in the maxillae and mandible (Table 6).

**Table 6:** Comparison between mean values FD of preoperative radiograph and mean values of bone densities from different sites FD: fractal dimension

	Mean FD values of preoperative radiograph	Mean bone densities values from different sites
Maxillary Molar	1.515	0.297
Maxillary Premolar	1.523	0.305
Mandibular Molar	1.550	0.379
Mandibular Premolar	1.563	0.489

## Discussion

Bone quality is a major determinant of implant prognosis [1]. It has been proven that good primary stability, and, consequently, a higher success rate, is found in implants inserted in bone of good quality such as in the mandible [16].

To evaluate the bone quality, Trisi and Rao [14] and Friberg et al. [17] performed bone biopsies and evaluated the bone quality through histomorphometric analyses. However, these methods are difficult to apply in a dental clinical environment. On the other hand, ISQ values are acceptable indicators to predict bone density and primary stability but this method cannot be used before the implant insertion.

In our study, bone specimens were harvested prior to implant placement and have been used as an absolute reference to compare the values of FD and ISQ.

The bone density was calculated using the mass to volume ratio. The results of bone densities were different when comparing maxillary recipient sites to mandibular sites. On the other hand, the bone densities in the maxillary posterior region were lower than in the mandibular posterior region, with mean values range between 0.301 and 0.434, respectively. This shows that the mass of bone cut out from the maxillae sites was less than the mandible despite the volume being calibrated for all sites.

Comparing ISQ values to the bone densities values, a positive significant correlation was shown in the posterior region of the mandible and the maxillae. To evaluate the quality and quantity of bone density in dental radiology, many studies have already been carried out using many methods such as densitometric and radiomorphometric measurements [18]. The bone density could be measured also by quantitative computed tomography [19], dual photon X-ray absorptiometry [20], or using conventional CT [21]. However, their role is limited due to the difficulty of equipment in the dental clinic or the high radiation dose and their unjustified use for a single implant.

In the oral and maxillofacial radiology field, FD, which is a method that quantifies the trabecular pattern by analyzing the bone density non-invasively, has been suggested [22]. In this concept, an FD analysis of periapical radiographs was proposed to assess alveolar bone patterns. A higher FD value indicates a more complex structure and, in some maxillary regions, showed a lower FD value, which directly relates to the reduction in the number of trabecular terminal points [23].

The results of our study showed FD values between 1.487 and 1.551 in the maxillary posterior region and between 1.5 and 1.614 in the mandibular posterior region. These significant results can be directly related to the reduction in the number of trabecular terminal points, whereas the FD values between pre and postoperative radiographs in all regions and sites showed no statistical difference, this concludes that the reorganization of the trabecular bone architecture after drilling and implant insertion was not verified.

Southard et al., in their study, reported a positive correlation between FD and bone density and suggested that the diminishing of FD corresponds to a reduction in bone density. Bone density and fractal dimension increase simultaneously [24]. Furthermore, Abdulhameed et al. concluded that implants with low FD values may indicate a decrease in stability [25].

FD values are not affected by conditions surrounding radiography such as the contrast of the image, tube angulation, and quantity of irradiation within an acceptable clinical standard. However, FD could have different results under other factors like image processing method and noise.

Bone anatomy in patients and the experimental method followed for the FD analysis can cause a variation in the results, which indicates that the FD should be used with certain restrictions.

In the current study, the reduction of the pixels of ROI, and the optimal range of box-counting were used to obtain the most accurate values in the estimation of bone density. The bone density of all specimens was calculated using the ratio of bone mass and volume. A significant correlation was found between bone densities and FD values in all implant sites in the maxillae and mandible of the posterior region using only the intraoral PSP radiographs.

It is important to focus on the fact that when the bone density value decreased, the mass of the bone biopsy diminished, thus the trabecular complexity was reduced, which leads to FD values being reflected and decreased.

Generally, high FD values are expected to be related to patients with high bone density, and this conclusion is similar to our study, indicating that the FD analysis from periapical radiographs, under a certain condition, can be used to estimate bone density prior to implant insertion. Tatli et al. noted a significant correlation between bone density values from CBCT and the ISQs values derived from RFA [26].

In this study, a statistically significant relationship was shown between implant primary stability and the fractal dimension in the mandibular posterior region, contrary to the maxillary posterior region, in which a low statically significant correlation was noted. It is clear to say that the trabecular pattern of the mandible could be seen in dense bone and the morphological analysis could be easier and greater significant values for FD, ISQ, and bone densities could be registered.

In order to apply the FD clinically as a tool of diagnosis, its calculation should be unified especially so that the FD values are not affected by the way in which the radiography was performed.

According to the results of the present study, the estimation of bone density prior to the implant placement in the recipient sites using FD based on digital periapical radiographs is a useful method for a surgical plan and a promising tool to predict bone density. Finally, our study is not without limitations. Because of the limited number of subjects investigated, definite conclusions must be delayed until future research with larger groups validates our findings.

## Conclusions

There is a significant correlation between the bone densities of the implant sites in the molar-premolar maxillary and mandibular sites, the FD analysis on digital periapical radiographs using the PSP technique, and the primary stability measured using resonance frequency analysis. A correlation was also found between FD and ISQ values in the posterior regions, and all executed analyses were helpful in estimating the bone density of implant placement using FD, offering complementary information about predictable bone density.

## References

[1] Esposito M, Hirsch JM, Lekholm U, Thomsen P (1998). Biological factors contributing to failures of osseointegrated oral implants, (II). Etiopathogenesis. Eur J Oral Sci.

[2] Shalabi MM, Wolke JG, Jansen JA (2006). The effects of implant surface roughness and surgical technique on implant fixation in an in vitro model. Clin Oral Implants Res.

[3] Meredith N, Alleyne D, Cawley P (1996). Quantitative determination of the stability of the implant-tissue interface using resonance frequency analysis. Clin Oral Implants Res.

[4] Mandelbrot BB (1983). The fractal geometry of nature (3rd ed).

[5] Weibel ER (1991). Fractal geometry: a design principle for living organisms. Am J Physiol.

[6] Badwal RS (1993). The application of fractal dimension to temporomandibular joint sounds. Comput Biol Med.

[7] Fazzalari NL, Parkinson IH (1996). Fractal dimension and architecture of trabecular bone. J Pathol.

[8] Updike SX, Nowzari H (2008). Fractal analysis of dental radiographs to detect periodontitis-induced trabecular changes. J Periodontal Res.

[9] Jolley L, Majumdar S, Kapila S (2006). Technical factors in fractal analysis of periapical radiographs. Dentomaxillofac Radiol.

[10] Tosoni GM, Lurie AG, Cowan AE, Burleson JA (2006). Pixel intensity and fractal analyses: detecting osteoporosis in perimenopausal and postmenopausal women by using digital panoramic images. Oral Surg Oral Med Oral Pathol Oral Radiol Endod.

[11] Zeytinoğlu M, İlhan B, Dündar N, Boyacioğlu H (2015). Fractal analysis for the assessment of trabecular peri-implant alveolar bone using panoramic radiographs. Clin Oral Investig.

[12] Soğur E, Baksı BG, Gröndahl HG, Sen BH (2013). Pixel intensity and fractal dimension of periapical lesions visually indiscernible in radiographs. J Endod.

[13] Mu TJ, Lee DW, Park KH, Moon IS (2013). Changes in the fractal dimension of peri-implant trabecular bone after loading: a retrospective study. J Periodontal Implant Sci.

[14] Trisi P, Rao W (1999). Bone classification: clinical-histomorphometric comparison. Clin Oral Implants Res.

[15] Misch CE (1990). Density of bone: effect on treatment plans, surgical approach, healing and progressive bone loading. Int J Oral Impl.

[16] Herrmann I, Lekholm U, Holm S, Kultje C (2005). Evaluation of patient and implant characteristics as potential prognostic factors for oral implant failures. Int J Oral Maxillofac Implants.

[17] Friberg B, Sennerby L, Roos J, Johansson P, Strid CG, Lekholm U (1995). Evaluation of bone density using cutting resistance measurements and microradiography: an in vitro study in pig ribs. Clin Oral Implants Res.

[18] Çağlayan F, Dağistan S, Keleş M (2015). The osseous and dental changes of patients with chronic renal failure by CBCT. Dentomaxillofac Radiol.

[19] Rosenthal DI, Ganott MA, Wyshak G, Slovik DM, Doppelt SH, Neer RM (1985). Quantitative computed tomography for spinal density measurement: factors affecting precision. Invest Radiol.

[20] Pouilles JM, Tremollieres F, Todorovsky N, Ribot C (1991). Precision and sensitivity of dual-energy x-ray absorptiometry in spinal osteoporosis. J Bone Miner Res.

[21] Norton MR, Gamble C (2001). Bone classification: an objective scale of bone density using the computerized tomography scan. Clin Oral Implants Res.

[22] Tores SR, Chen CS, Leroux BG, Lee PP, Hollender LG, Schubert MM (2011). Fractal dimension evaluation of cone beam computed tomography in patients with biophosphonate-associated osteonecrosis. Dentomaxillofac Radiol.

[23] Ruttiman UE, Webber RL, Hazelrig JB (1992). Fractal dimension from radiographs of peridental alveolar bone: a possible diagnostic indicator of osteoporosis. Oral Surg Oral Med Oral Pathol.

[24] Southard TE, Southard KA, Krizan KE, Hillis SL, Haller JW, Keller J, Vannier MW (2000). Mandibular bone density and fractal dimension in rabbits with induced osteoporosis. Oral Surg Oral Med Oral Pathol Oral Radiol Endod.

[25] Abdulhameed EA, Al-Rawi NH, Uthman AT, Samsudin AR (2018). Bone texture fractal dimension analysis of ultrasound-treated bone around implant site: a double-blind clinical trial. Int J Dent.

[26] Tatli U, Salimov F, Kurkcu M, Akoglan M, Kurtoglu C (2014). Does cone beam computed tomography-derived bone density give predictable data about stability changes of immediately loaded implants?: a 1-year resonance frequency follow-up study. J Craniofac Surg.

